# Clinical adoptive regulatory T Cell therapy: State of the art, challenges, and prospective

**DOI:** 10.3389/fcell.2022.1081644

**Published:** 2023-01-30

**Authors:** Leila Amini, Jaspal Kaeda, Enrico Fritsche, Andy Roemhild, Daniel Kaiser, Petra Reinke

**Affiliations:** ^1^ Berlin Center for Advanced Therapies, Charité-Universitätsmedizin Berlin, Berlin, Germany; ^2^ Berlin Institute of Health—Center for Regenerative Therapies, Charité-Universitätsmedizin Berlin, Berlin, Germany

**Keywords:** adoptive regulatory T cell therapy, immune regulation, regenerative medicine, advanced medicinal therapeutic products, clinical trials

## Abstract

Rejection of solid organ transplant and graft *versus* host disease (GvHD) continue to be challenging in post transplantation management. The introduction of calcineurin inhibitors dramatically improved recipients’ short-term prognosis. However, long-term clinical outlook remains poor, moreover, the lifelong dependency on these toxic drugs leads to chronic deterioration of graft function, in particular the renal function, infections and *de-novo* malignancies. These observations led investigators to identify alternative therapeutic options to promote long-term graft survival, which could be used concomitantly, but preferably, replace pharmacologic immunosuppression as standard of care. Adoptive T cell (ATC) therapy has evolved as one of the most promising approaches in regenerative medicine in the recent years. A range of cell types with disparate immunoregulatory and regenerative properties are actively being investigated as potential therapeutic agents for specific transplant rejection, autoimmunity or injury-related indications. A significant body of data from preclinical models pointed to efficacy of cellular therapies. Significantly, early clinical trial observations have confirmed safety and tolerability, and yielded promising data in support of efficacy of the cellular therapeutics. The first class of these therapeutic agents commonly referred to as advanced therapy medicinal products have been approved and are now available for clinical use. Specifically, clinical trials have supported the utility of CD4^+^CD25+FOXP3+ regulatory T cells (Tregs) to minimize unwanted or overshooting immune responses and reduce the level of pharmacological immunosuppression in transplant recipients. Tregs are recognized as the principal orchestrators of maintaining peripheral tolerance, thereby blocking excessive immune responses and prevent autoimmunity. Here, we summarize rationale for the adoptive Treg therapy, challenges in manufacturing and clinical experiences with this novel living drug and outline future perspectives of its use in transplantation.

## 1 Introduction

Success of solid organ transplantation (SOT) and allogenic haematopoietic stem cell transplantation (HSCT) is dependent upon dampening the recognition of and reaction to “foreign” allogenic cells by the exquisitely designed human immune system. The inability to modulate alloantigen recognition leads to deterioration in graft function with poor quality of life and ultimately graft rejection, i.e., loss of the transplanted organ, or fatal graft versus host disease (GvHD). With the aim of disrupting the injurious sequence of events, pharmacologic immunosuppressants have been introduced into the clinical management of SOT. The immunosuppressive agents include proliferation inhibitors (e.g., mycophenolate and azathioprine), glucocorticoids (e.g., Prednisone), mammalian target of Rapamycin (mTOR) inhibitors (e.g., Sirolimus and Everolimus) as well as Calcineurin inhibitors (e.g., Tacrolimus and Cyclosporine A) (Taylor et al., 2005). Often these are administered in combination with biological products, such as antibody preparations, either as pre-induction therapy or to manage acute conditions (e.g., Alemtuzumab, Basiliximab or anti-thymocyte globulin) (Taylor et al., 2005). This approach frequently leads to a broad range of adverse effects, including nephrotoxicity, *Diabetes mellitus* type II, oedema, neurotoxicity, hypertension, haematological cytopenia, skin disorders *etc*. Furthermore, patient compliance and adherence is often poor, especially if multiple classes of drugs are prescribed (Chisholm, 2002). As a consequence efficacy is diminished leading to substantially reduced quality of life (Madariaga et al., 2016). Relative to SOT recipients the level of immunosuppression prescribed to post HSCT subjects is less intense. This difference is attributable to the tolerance induction mechanisms associated with transplanted stem cell progeny, however, the pre-existing memory cells within the transplant represent a reservoir of cells with the potency to induce life threatening GvHD. An additional concern associated with IS post HSCT in cancer treatment is the suppression of the graft versus leukaemia (GVL) effect.

The adverse effects and the poor quality of life associated with current intensive IS highlight the need for an alternative approach to improve long-term graft survival and facilitate reduction or even replace the toxic long-term application of pharmacological immunosuppressants. A range of cell types with immunoregulatory and regenerative characteristics have been sought as potential therapeutic option. The pre-clinical data from kidney (Siepert et al., 2012), heart (Ma et al., 2008), skin (Issa et al., 2010) transplant and GvHD (McDonald-Hyman et al., 2016) models show the potential benefits of adoptive Treg therapy. In particular, CD4^+^CD25^+^FOXP3^+^ Tregs have seen a rapid transfer from pre-clinical studies to Phase I/II clinical trials. The early clinical trials data have demonstrated safety, tolerability and potential of efficacy of the adoptive Treg therapy.

This report concentrates exclusively on human Treg studies. This review summarises functional human Treg data, providing the rationale for their application in humans, the manufacturing procedures, including published and ongoing clinical trials involving adoptive Treg therapy with a focus on SOT and HSCT recipients.

## 2 Functional characteristics of human regulatory T cells

### 2.1 FOXP3; The controller and beyond

Sakaguchi *et al.*, in 1985 published data showing a subset of CD4^+^ T lymphocytes constitutively expressing IL-2 receptor α (IL-2Rα) chain, CD25, suppressed autoimmune disease in thymectomised mice (Sakaguchi et al., 1985). Subsequently, numerous studies confirmed CD4^+^CD25^+^ T cells suppressed autoimmunity and that the lineage defining transcription factor Forkhead Box P3 (FOXP3) in collaboration with the nuclear factor of activated T cells (NFAT) controlled the expression of genes, which characterize Tregs (Wu et al., 2006). It is now widely accepted that FOXP3 orchestrates Treg development and function. Moreover, it was the FOXP3 inactivating mutations which confirmed the important role this key transcription factor plays in modulation of the immune system *via* the Tregs (Bennett et al., 2001). The mutated FOXP3 leads to the ‘scurfy’ phenotype in mice and the development of severe X-linked severe autoimmunity, immune-dysregulation poly-endocrinopathy enteropathy x-linked (IPEX) syndrome in humans (Bennett et al., 2001; Chang et al., 2005). The symptoms of IPEX syndrome include severe diarrhea, diabetes, skin conditions (such as eczema, erythroderma, or psoriasis), and thyroid disease (thyroiditis). Mutated FOXP3 is also associated with causing other severe autoimmune disorders, such as inflammatory bowel diseases (Okou et al., 2014) and allergy accompanied by increased IgE levels (Torgerson et al., 2007). Apart from FOXP3, point mutations mapping to *STAT5B* (Majri et al., 2018), *CTLA4* (Schubert et al., 2014) and *LRB* genes (Alangari et al., 2012) expressed by Tregs also lead to the development of autoimmune phenotypes. While the mutated genes would be expected to disrupt Treg function and present as autoimmune phenotype, a number of autoimmune disorders have also been reported to be associated with decreased Treg numbers in circulation (von Spee-Mayer et al., 2016; Viisanen et al., 2019). While these reports are controversial, they non-etheless imply reduced Treg numbers can precipitate immunological pathologies. For example, chronic rheumatoid arthritis is a systemic autoimmune disease leading to unchecked inflammation and tissue destruction in the joints, the data available imply these patients have reduced Treg numbers in circulation and the synovial fluid (Leipe et al., 2005). Similarly, the inflammatory autoimmune disease ankylosing spondylitis, which is associated with HLA-B27, is linked to reduced Treg numbers (Simone et al., 2021).

There is substantial evidence, Tregs, defined by expression of CD4, CD25 and FOXP3, are critical to immune mechanisms of tolerance maintaining immunological homeostasis. FOXP3 regulates Treg development, function and stability, while CD25 expressed in the absence of the IL-7 receptor α-chain (CD127) is used to better define Tregs (Yu et al., 2012). Furthermore, FOXP3 expression inversely correlates with CD127 expression (Yu et al., 2012). While an array of markers is used to define this highly heterogenous Treg lineage, it is generally classified into two groups, thymic-derived, central or natural Tregs (tTregs/nTregs) and induced or peripheral Tregs (iTregs/pTregs) (Dhamne et al., 2013; Povoleri et al., 2013). The latter develop following activation in presence of TGFβ combined with other factors such as IL-2 (Schmidt et al., 2016). There are no definitive markers to distinguish between the two subgroups, but there are epigenetic differences, i.e., a demethylated Treg-specific demethylation region (TSDR), which confers intrinsic instability on iTreg or pTreg in inflammatory and/or stress environment (Polansky et al., 2008). Nevertheless, there are further differences beyond TSDR demethylation as a targeted demethylation of the TSDR does not confer Treg functionality (Kressler et al., 2020). Usually, iTreg described *in vitro* generated Treg and pTreg are those induced in the periphery of the human body, outside the thymus (Shevach and Thornton, 2014). Human Tregs can be further sub-classified according to their level of differentiation, as naïve, effector memory, central memory or effector Treg (Shevyrev and Tereshchenko, 2019).

### 2.2 Suppressive mechanisms

**FIGURE 1:**
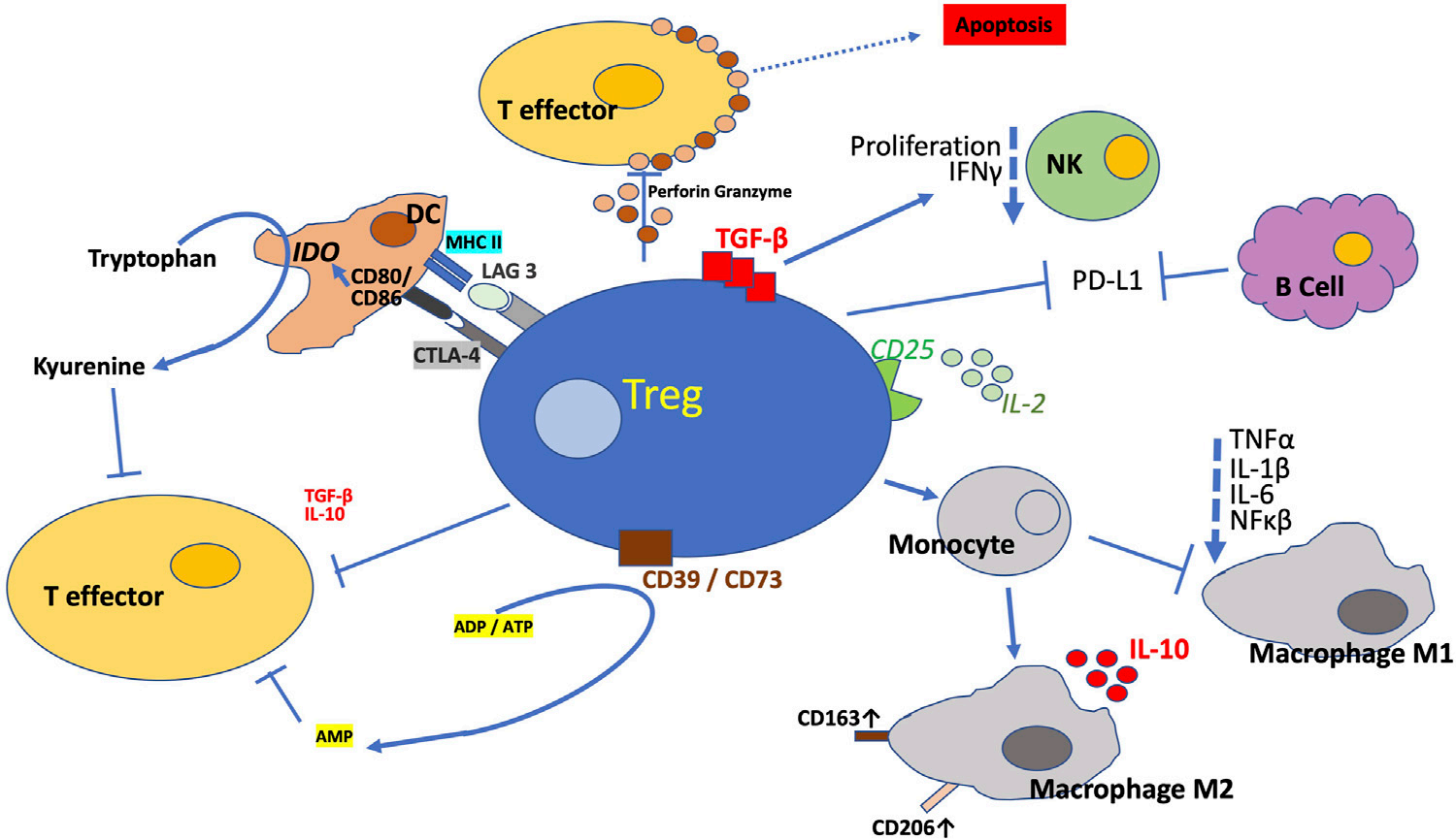
Mechanisms of Suppression. Tregs suppress innate and adaptive immune responses *via* myriad of direct and in-direct mechanisms to promote tolerance and dampen excessive deleterious immune response. The figure illustrates the multitude of mechanisms described *via* which Tregs exquisitely conducts the immune system. Treg synthesize a number of anti-inflammatory cytokines, this includes IL-10 and TGF-β and thereby modulating pro-inflammatory functions. Tregs block (co-)stimulation by antigen presenting cells *via* CTLA-4 and LAG3. Tregs induce apoptosis of target cells by releasing perforin and granzyme or *via* FasL expression. Tregs counteract pro-inflammatory signalling *via* ectoenzymes CD39 and CD73 which combine to dephosphorylate ATP and ADP to AMP and also through capture of IL-2 by the constitutively high expression of the IL-2 receptor α-chain, CD25. Tregs also directly impede Natural Killer cells *via* membrane bound TGF-β. Among the numerous mechanisms, Tregs are additionally reported to inhibit B cells *via* PDL-L1/PD-L2 PD-1 interaction. CTLA-4 blocks co-stimulation reducing CD80/CD86 expression and this triggers IDO upregulation. The regulatory T cells direct the differentiation of monocytes to M2 macrophages and so prevent their differentiation to M1 macrophages, which are pro-inflammatory. The Tregs also neutralise MHC II so that it is not available to the pro-inflammatory T cells. All this is ultimately controlled by increased expression of FOXP3, the principal orchestrator of Treg proliferation and stability through transcription of a number of genes. These genes promote the Treg programme *via* the numerous signal transduction pathways.

The precise details of Treg suppressive mechanisms remain equivocal, available data imply the CD4^+^CD25^high^ FOXP3^+^ lineage suppresses inflammatory processes *via* a multitude of mechanisms, as outlined in Figure 1. These include direct cell to cell contact, involving cell-surface specific receptors and secretion of inhibitory cytokines such as IL-10 and TGFβ, as well as modulation of extracellular factors impacting upon a great variety of immunological players. The multifaceted Tregs can release IL-10, a cytokine reported to be a key immunoregulator during viruses, bacteria, fungi, protozoa, and helminths infections, ameliorating the excessive Th1 and CD8^+^ T cell responses (Fujio et al., 2017). Observations suggest Tregs enhance efferocytosis by macrophages, which is a critically important effector arm of inflammation resolution (Proto et al., 2018). This is achieved *via* IL-10 signalling leading to increased phagocytic activity and CCL18 production, upregulated CD163 and downregulates HLA-DR in M2 macrophages as well as suppressing the release of TNFα and IL-6 (Tiemessen et al., 2007). IL-10 producing Treg may be of importance for mucosal tolerance in the human gut (Bakdash et al., 2015). Reduction of TGFβ and Treg in blood was found in Lupus nephritis patients (Xing et al., 2012). IL-10 and TGF-β secretion by Treg was also shown to inhibit pro-inflammatory cytokine secretion by CD4^+^ effector T cells in the context of cervical cancer (Adurthi et al., 2012). Surface TGF-β, which is predominantly expressed by CD45RO^+^ memory Tregs (de Jong et al., 1994), can suppress Natural Killer cell effector functions and downregulate the NKG2D receptor (Ghiringhelli et al., 2005). Human Treg secrete latent TGF-β (Levings et al., 2002), which binds to glycoprotein A repetitions predominant (GARP) on their surface (Stockis et al., 2009) and can be activated by αVβ8 integrin dimers expressed by activated Tregs and thereby suppress pro-inflammatory cells (Stockis et al., 2017).

In addition, Tregs can mediate the induction of M2 macrophages in a cytokine independent (Tiemessen et al., 2007), contact dependent manner (Bayry et al., 2007). Another immunosuppressive characteristic of Tregs is the shedding of soluble TNFα receptor II, which can neutralize TNFα and thus prevent pro-inflammatory signalling (van Mierlo et al., 2008).

Dendritic cells, a diverse family of cells, play a crucial role in linking our innate and adaptive immune system to initiate adequate T cell responses. They function by acquiring, processing and presenting antigens to T cells. Dendritic cells continually present self and non-self- antigens, thereby promote tolerance *via* control of Tregs. Integrin αvβ8 on dendritic cells can activate TGF-β, a process that can contribute to the development of pTregs, especially in tumours (Seed et al., 2021). Disruption of dendritic cell-expressed TGFβ receptor (TGFβR) impairs the tolerogenic function of dendritic cells and fosters autoimmunity (Ramalingam et al., 2012). Increased dendritic cell numbers are accompanied by a concomitant increase in Tregs, paradoxically, elimination of Tregs leads to an increment of dendritic cells in numbers, suggesting these lineages jointly regulate homeostasis of each other, probably mediated by indoleamine 2, 3-dioxygenase (Wu et al., 2018).

Equally, the high expression of CD25 by Tregs facilitates the capture of IL-2, thereby depriving their competitors, the effector T cells of this cytokine, an important mechanism by which immune suppression is mediated (Barthlott et al., 2005). IL-2 is essential for proliferation and differentiation of effector T cells and Tregs. Furthermore, Tregs bind peptide-major histocompatibility complex class II (MHCII)-peptide complexes specific to their TCR from the surface of antigen presenting cells (APCs), ingest and degrade them *via* trans-endocytosis. (Akkaya et al., 2019). Thus, Tregs indirectly prevent an activation of effector T cells by reducing the ability of APCs to present specific antigens (Akkaya et al., 2019).

### 2.3 Co-inhibitory receptors

Counter to co-stimulatory receptors, Treg express antigens which are classified as co-inhibitory receptors. Among the most studied surface CD4^+^ Treg molecules that exert immunosuppressive effects is cytotoxic T-lymphocyte-associated protein 4 (CTLA-4; CD152) (Birebent et al., 2004; Pedroza-Gonzalez et al., 2015). CTLA-4 diminishes proliferation of alloantigen-specific effector T cells (Birebent et al., 2004) and decreases IFNγ production by effector T cells (Flores-Borja et al., 2008). These observed changes are triggered by the downregulation of co-stimulatory molecules CD80 and CD86 (Wing et al., 2008), which are captured from the surface of APC *via* trans-endocytosis followed by their degradation (Qureshi et al., 2011). Disruption of the Treg CTLA-4 pathway leads to increased internalization of CTLA-4 in rheumatic arthritis patients (Flores-Borja et al., 2008). A myriad of critical immune system control mechanisms recognizes self and non-self, thus blocking indiscriminate damage to tissues or inappropriate immune responses. Immune checkpoints include binding of specific partners/antigens to T cell surface proteins, thereby inhibiting T cell activation and prevent elimination of cells expressing the antigens. However, the multitude of check points are frequently co-opted by tumour cells to evade the immune systems and so avoid eradication. Within the last decade immune checkpoint blockade therapies targeting CTLA-4 have been developed to treat cancer patients. For example, ipilimumab (anti-CTLA-4) is an approved ICB therapy for metastatic melanoma (Hodi et al., 2010). Similarly, ICB therapies, e.g., nivolumab, have been approved targeting programmed death (PD)-1/PD-Ligand (L), for example in the treatment of lung cancer (Rittmeyer et al., 2017). Reported data show human CD4^+^ Treg express PD-L1 (B7 homolog 1 or CD274) in response to IL-7 (Cristelli et al., 2018) and as a consequence induce apoptosis in PD-1 expressing cells such as effector T cells (Dong et al., 2002) or autoreactive B cells (Gotot et al., 2018). Similarly, another immune checkpoint receptor Lymphocyte-activation gene 3 (LAG3; CD223) promotes immunosuppression by negatively regulating proliferation and blocking activation of Treg. LAG3 binds to MHCII molecules with much higher affinity and thus outcompetes CD4^+^ effector T cells (Huard et al., 1997). However, it is suggested that LAG3 functions entirely independent of CD4 (Wang et al., 2019). Published data suggest LAG3 functions through down regulating both the activation and pro-inflammatory action of human dendritic cells (Bayry et al., 2007). Similarly, a recently identified regulatory mechanism, the T cell immunoglobulin and immunoreceptor tyrosine‐based inhibitory motif (ITIM) domain (TIGIT) is a co‐inhibitory receptor expressed by activated T cells, Treg, and NK cells. Similar to CTLA‐4 and CD28, TIGIT competes with its costimulatory counterpart CD226 for the same ligands (CD155 and CD112) and mediates immune suppression in tumours and chronic infections (Fuhrman et al., 2015).

### 2.4 Apoptosis and anti-inflammatory mediators

A more direct form of immune regulatory mechanisms is induction of apoptosis. Treg cell contact with a respective target cell, e.g., CD8^+^ T cells, induces apoptotic signalling pathways *via* induction of the Fas-Fas Ligand (FasL) axis, i.e., members of the tumor necrosis factor (TNF)-receptor and TNF family, respectively (Strauss et al., 2009). The ligation of Fas with FasL results in the activation of a caspase cascade that initiates apoptosis (Strauss et al., 2009). Apoptosis is also activated *via* the perforin/granzyme pathway deployed by the Treg to target and kill cells, e.g., CD4^+^ and CD8^+^ effector T cells, monocytes, neutrophils and dendritic cells, accompanied by adhesion *via* CD18 (Grossman et al., 2004; Lewkowicz et al., 2006).

Tregs can attune the extracellular environment to promote anti-inflammatory activity *via* the CD39 and CD73 ecto-enzymatic signal transduction, which dephosphorylates adenosine di- and tri-phosphate (ADP and ATP) to yield extracellular adenosine mono-phosphate (AMP). The latter impedes activation, proliferation, cytokine synthesis and cytotoxicity of T cells. ADP and ATP are released by injured or necrotic cells causing pro-inflammatory signalling *via* purinergic P2 receptor activation (Bours et al., 2006). CD39 is also known as ectonucleoside triphosphate diphosphohydrolase-1 or ENTPD1 and is expressed by effector/memory FOXP3^+^ Tregs (Borsellino et al., 2007). CD39 acts as an immunosuppressant in co-ordination with membrane bound glycoprotein CD73, also known as ecto-5′-nucleotidase or NT5E (Nagate et al., 2021). Data imply CD73 can be either co-expressed on Treg or presented on neighbouring cells or exosomes (Schuler et al., 2014). CD39 is also expressed by adult T cell leukaemia/lymphoma and it is overexpressed in a number of neoplasms (Nagate et al., 2021). Furthermore, data show a majority of adult T cell leukaemia/lymphoma cells which share the Treg phenotype, CD4^+^CD25^+^CCR4^+^FOXP3^+^, leading to the suggestion that adult T cell leukaemia/lymphoma cells are descendants of Tregs (Kohno et al., 2005). But this and the suggestion adult T cell leukaemia/lymphoma cells are immunosuppressive is equivocal. CD39 is also reported to be clinically relevant for acceptance of kidney grafts (Durand et al., 2018). It is suggested that the full therapeutic potential of Tregs is conferred by cells co-expressing CD39 and CD73 (Grist et al., 2018).

Activation of chemotaxis pathways directs Tregs to the site of interest to effect their immunosuppressive activity, e.g., high expression of CCR4 directs Tregs to mature dendritic cells, which produce the respective ligands (Birebent et al., 2004). Tregs are a heterogenous population of T cells specialized for distinct suppressive tasks: For example, Bcl-6^+^ CXCR5^+^ are referred to as follicular Tregs and can suppress germinal center reactions (Chung et al., 2011), e.g., by inhibition of follicular helper T cells (Miles et al., 2015). Moreover, tumor infiltrating Tregs express CCR8 (De Simone et al., 2016) and Tregs with T cell receptors specific to antigens only present in a distinct tissue show tissue-specific homing potential, e.g., air-borne antigen-specific Tregs comprise a lung-homing signature (Bacher et al., 2016). Indeed, tissue-specific methylation patterns were identified for tissue-resident Tregs (Delacher et al., 2017).

All these functional attributes qualify human Treg as an attractive candidate for application in transplantation in hyperinflammatory immune disorders. Indeed, Treg have been associated with operational tolerance induction after SOT (Taubert et al., 2016), severity of GvHD (Matsuoka et al., 2013) and are reportedly reduced in autoimmune disorders (von Spee-Mayer et al., 2016; Viisanen et al., 2019), which displays the rational for applying them to different indications.

## 3 Manufacturing of human regulatory T cell products

**FIGURE 2:**
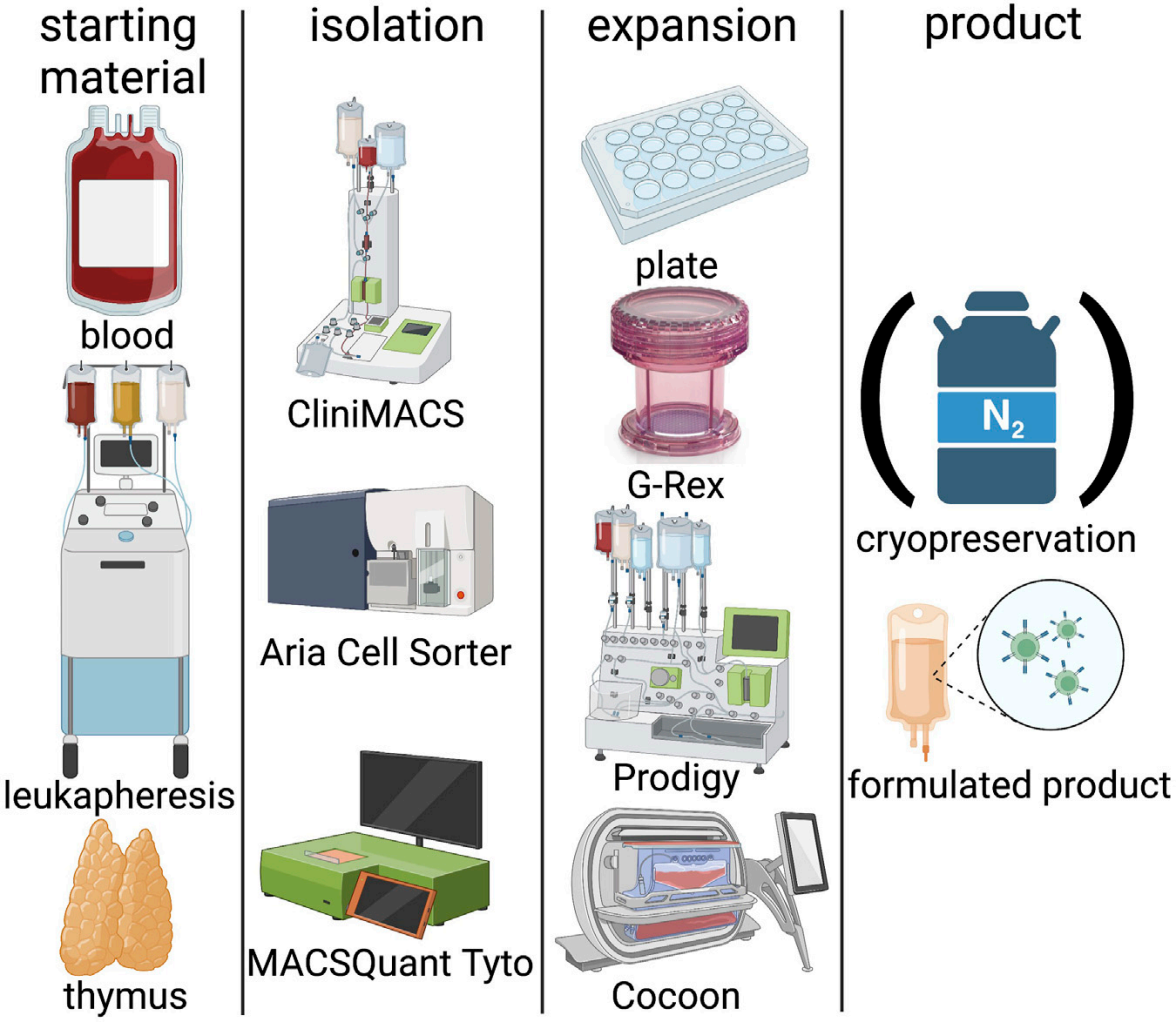
Technologies in Treg manufacturing. Blood, leukapheresis or thymi can be used as starting material. Isolation is classically performed by CliniMACS, Aria cell sorter or the more recent and GMP compliant MACSQuant Tyto system. Expansion systems include classical 24-well plates, semi-closed GRex bioreactors, or fully closed systems such as the Prodigy or Cocoon platforms. Treg products can be either frozen in liquid nitrogen or applied freshly. (Created with BioRender).

Two major challenges must be overcome to move from bench to bedside and realise the therapeutic potential of Tregs; lack of Treg specific phenotype and the limited number of these cells in circulation. Tregs represent only 1%–5% of total CD4^+^ T cells, therefore *ex-vivo* expansion, typically over a period of 2–5 weeks, is crucial to obtaining sufficient cell numbers for clinical application (Chandran et al., 2017; Wiesinger et al., 2017; Fraser et al., 2018; Landwehr-Kenzel et al., 2018; Mathew et al., 2018; Roemhild et al., 2020). There is a considerable heterogeneity among the reported isolation and expansion protocols with varying levels of purity and proliferation (Figure 2). Autologous products are preferable for early clinical trials, as they are more likely to be tolerated long term by the recipient, although this approach is not suited for mass production and associated with challenges for commercialization. Nevertheless, autologous T cell products have already made it to the market despite high pricing and logistic challenges. However, as Tregs must be manufactured for each patient, the GMP compliant manufacturing must be robust to ensure reproducibility, which can be challenging as the underlying pathology, this will include subclinical factors, which may affect immune cell numbers, proliferation and functional characteristics. Technologies used in manufacturing of clinical grade Treg products are summarized in Figure 2. The manufacturing of autologous Treg products invariably on occasions includes highly morbid and sometimes elderly subjects may raise concerns regarding product generation complications e.g., inflammaging, which is associated with a decrease in Treg numbers (Zhou et al., 2022), their potency (Hwang et al., 2009) and increasing immune cell senescence (Schaenman et al., 2018). However, our studies demonstrated the percentages of Treg in the circulation among kidney transplant recipients between 18 and 87 years of age were largely unchanged (Landwehr-Kenzel et al., 2018). Although their maturation may be compromised, successful generation with acceptable functionality of Treg products from such end-stage renal disease patients is possible (Landwehr-Kenzel et al., 2018). As far as we can ascertain there are no authoritative data regarding the impact of aging microenvironment on adoptively transferred Treg administered to geriatric transplant recipients. Indeed, in general pharmacokinetics and metabolism of systemically administered viable polyclonal Tregs are ill-defined as they are phenotypically indistinguishable from Tregs in general circulation unless specifically labelled (Oo et al., 2019).

### 3.1 Isolation

It is widely accepted that CD4, CD25 and FOXP3 are key Treg markers and are partially associated with their functional properties. Tregs can be isolated from the peripheral blood (PB) (Landwehr-Kenzel et al., 2018; Roemhild et al., 2020), umbilical cord (Brunstein et al., 2011; Brunstein et al., 2016), thymus (Romano et al., 2021) or leukapheresis products (Peters et al., 2008; Wiesinger et al., 2017; Mathew et al., 2018). Of the available options PB is the least invasive, well tolerated by the patient and routinely collected in the clinics. However, sampling containers for small amounts of blood are currently not specifically designed and labelled suitable for cell product manufacturing, which can make acquisition of manufacturing licenses challenging. However, the volume of PB required can be a limiting factor and therefore leukapheresis is the more common source of starting material. Although leukapheresis is highly invasive and associated with risk, it can yield Tregs in sufficient numbers and purity.

The initial isolation of the Treg starting material is commonly achieved using a closed system, for example CliniMACS® for magnetically activated cell sorting (MACS) (Peters et al., 2008; Brunstein et al., 2016; Wiesinger et al., 2017; Landwehr-Kenzel et al., 2018; Mathew et al., 2018) or the latest fluorescently activated cell sorting (FACS) technology, MACSQuant® Tyto®, which yields high purity of CD4^+^CD25^+^CD127^low^ Tregs from peripheral blood mononuclear cells (PBMC) (Au et al., 2019). MACS isolation procedures yield purities of 40%–60% in the starting population (Peters et al., 2008). The isolation strategies for CD4^+^ Tregs vary using a combination of positive and negative selection, from limited to only CD25 enrichment (Brunstein et al., 2011; Brunstein et al., 2016), to CD8 depletion followed by CD25 enrichment (Landwehr-Kenzel et al., 2018; Roemhild et al., 2020) through to post expansion selection on day 14; CD8, CD19, CD127 depletion and CD25 enrichment or CD25 enrichment with subsequent CD8 and CD19 depletion (Peters et al., 2008; Mathew et al., 2018) (Wiesinger et al., 2017). Although not universally accepted by international regulatory authorities, investigators have reported using an open system (FACSAria^TM^ II) for isolation of CD4^+^CD127^low/−^CD25^+^ T cells for clinical product generation in early phase clinical trials (Putnam et al., 2008; Bluestone et al., 2015). The exclusion of cells expressing the IL-7 receptor*, i.e*. CD127 on T cells has been suggested to further optimise the Treg isolation purity as it inversely correlates with FOXP3 expression (Liu et al., 2006). The latter is intracellular and therefore methods designed to detect would disrupt the cell and therefore counteract expansion efforts. Reported data imply IFNγ producing CD8^+^ T cells are associated with a poor outcome post-transplant (Donckier et al., 2009); therefore, protocols are designed to exclude these cells from the Treg product. Therefore, usually CD8^+^ T cells are excluded from the starting material by a negative selection step. An efficient CD8^+^ depletion step yields a pure population of Tregs and ensures the former do not proliferate during manufacturing culture processes (Landwehr-Kenzel et al., 2018; Roemhild et al., 2020) (Peters et al., 2008) (Mathew et al., 2018) (Wiesinger et al., 2017).

### 3.2 Expansion

A major obstacle to expansion is the terminal differentiation of Tregs, requiring stimulation to proliferate. A wide range of reagents and protocols have been described to achieve a clinically useful dose of cells. The most commonly used activation method mimics antigen presenting cells (APC) *in-vitro* by using CD3/CD28 antibody-coated microbeads that bind to the T cell receptor complex and co-stimulatory receptors expressed by Tregs. Clearly the Tregs must be separated, by magnetic extraction, from the microbeads prior to cell infusion (Roemhild et al., 2020).

Tregs are generally expanded in presence of IL-2 and rapamycin (Peters et al., 2008; Brunstein et al., 2011; Wiesinger et al., 2017; Landwehr-Kenzel et al., 2018; Mathew et al., 2018) to maximise yield and purity of Treg therapeutic products. This approach can lead to several 1000-fold expansion (Roemhild et al., 2020). The concentration and the timing of when IL-2 is added to yield optimal Treg expansion is equivocal, varying from 100 to 1000 IU/ml. Secondly, rapamycin preferentially inhibits the proliferation and function of CD25^−^ conventional effector T cells (Battaglia et al., 2006), thereby enhances Treg expansion and functionality of Treg products (Fraser et al., 2018). Data imply this is not entirely due to rapamycin acting directly on Tregs, but because the effector T cells have higher sensitivity to this IS agent, as a consequence the Treg: effector T cell ratio is enhanced (Strauss et al., 2007). These observations are supported by a large body of data. It is reported the rapamycin may increase the Treg CD25 expression and maintain FOXP3 expression throughout the duration of the culture (Safinia et al., 2016). Therefore, overall addition of rapamycin is expected to yield a purer and more potent therapeutic agent. Furthermore, to avoid Tregs differentiating into highly proinflammatory Th17 cells, that lack suppressive activity, TGFβ is frequently included in the culture medium (Mathew et al., 2018).

The level of expansion can also be influenced by the culture media (e.g., X-VIVO^TM^15; TexMACS^TM^; RPMI-1640), composition of which vary, e.g., inclusion of fetal bovine serum (Landwehr-Kenzel et al., 2018), human serum (Bluestone et al., 2015; Mathew et al., 2018) or autologous plasma (Wiesinger et al., 2017). Inclusion of human serum is usually preferred by regulatory authorities over xenogeneic products, but apart from being expansive, the inter-product variability is of some concern. As alluded above, some clinical trials completely avoid expansion and infuse freshly isolated Tregs (Di Ianni et al., 2011; Martelli et al., 2014; Meyer et al., 2019). But this approach may not always be feasible for the cells to be efficacious, the number of cells must exceed the physiological levels. Given the patients are often frail with concomitant pathologies, this option might not be available to all subjects. Currently, investigators try to develop serum-free expansion for GMP grade Treg products to overcome ethical and safety concerns regarding the use of serum (MacDonald et al., 2022).

Apart from the agents for the agents used to expand Treg products, also adjustment of the cell density is a critical factor to optimize expansion rates and quality cell products (MacDonald et al., 2022). Furthermore, expansion is possible in classical cell culture plates, but also semi-closed bioreactors (Chakraborty et al., 2013; Roemhild et al., 2020). More recently, technological developments have the potential to automate manufacturing of ATMPs for example using the CliniMACS Prodigy® system to generate a clinical grade Treg product, which helps standardization (Marín Morales et al., 2019). Furthermore, the use of a closed system minimizes the risk of contaminations. Applications of the multi/tool semi-automated systems permit streamlining the manufacturing process and thereby generating clinical grade ATMPs with increased safety and efficiency as well as a higher grade of standardization (Fritsche et al., 2020).

### 3.3 Cryopreservation

The manufacturing of cellular therapeutics is complex with challenges distinct from the conventional medical therapies. The cells are collected from living or deceased donor and subjected to selection, expansion, sometimes including genetic modifications, prior to administration to the recipient. This supply chain imposes logistical challenges from obtaining the starting material through to manufacturing and delivery must be co-ordinated such that cells remain viable and functional throughout the whole supply chain. Absence of effective, efficient cell preservation and cost considerations have restricted rapid growth in cellular therapeutics, apart from GMP compliant challenges. Efficient and effective cryopreservation enables co-ordination of the therapy for the recipient and GMP compliant manufacturing. Cryopreservation has advantages, enabling long-term storage, flexibility of the timing of infusion, the option to ship the product and more time to perform release analysis to ensure safety and functionality. It allows centralized manufacturing whereas fresh products in general have to be manufactured within short distance to the centre for clinical application of the cells. However, the universally and widely established cryoprotectant, i.e. Dimethyl Sulfoxide (DMSO) is associated with toxicities to the patients. These include allergies, gastrointestinal, cardiac and neurological complications to patients infused with DMSO cryopreserved cell therapy products (Galvao et al., 2014). Despite the efforts to improve and understand freezing and thawing processes, cryopreservation of cells is still generally dependent on DMSO as the cryoprotectant This is largely because DMSO is efficient in protecting cells intracellular integrity. The T cell cryopreservation media traditionally includes 10% DMSO, this can be reduced to 5%, with human serum albumin, foetal bovine serum, human AB serum with addition of sugars and small molecules (Kaiser et al., 2021). Apart from the cost considerations, this adds another layer of GMP compliant complexity to manufacturing ATMP products. Efforts to reduce or eliminate DMSO have in general failed to be universally acceptable, because lack of benefits across different cell types. The Biomedical Excellence for safer Transfusion Collaborative Group in 2017 reported T cells to be more sensitive to cryopreservation than other cell types, e.g., haematopoietic stem cells (Worsham et al., 2017). Indeed, DMSO has an adverse effect on Tregs in that they exhibit reduced viability, abnormal cytokine secretion and it compromises surface marker expression (Florek et al., 2015; Golab et al., 2018). Therefore, the option of Treg cryopreservation is challenging in terms of viability and recovery, more so as data imply CD25 and FOXP3 expression, and thus the suppressive activity is impaired after thawing (Golab et al., 2018). As a consequence, Tregs may need to be re-stimulated to reinvigorate the full suppressive potential after thawing (Peters et al., 2008; Sadeghi et al., 2013; Luo et al., 2017). Investigators have explored replacing DMSO with a wide range of alternatives, focusing on small molecules, sugar or protein-based products. These included using glycerol or glucose (Smit Sibinga and Meryman, 1990; Lusianti et al., 2013; Shu et al., 2014; Capicciotti et al., 2015; Rogers et al., 2018). But at high concentrations glycerol is also toxic and has to be removed prior to infusion (Lusianti et al., 2013; Shu et al., 2014; Capicciotti et al., 2015). Other sugars have also been tried for a varied cells types (Brodthagen et al., 1985; Gook et al., 1999; Deller et al., 2015), however literature search implies no single cryopreservation formulation was effective across all cell types has been described. Therefore, it would be expedient to develop a specific cryoprotectant suitable for Treg that is avoid of DMSO. In summary, despite the considerable efforts to identify cryoprotectant for T or NK cells which exclude DMSO have largely failed. This is a source of concern given the rapid increase in new generation of cellular clinical therapeutics which ideally need a cryopreservation option of product that can be directly infused, is safe and void of adverse functional effects of the T cells. Nevertheless, promising protocols have been developed which may ease clinical application and accessibility of Treg products (Kaiser et al., 2021). In general, manufacturing technologies of ATMPs, including Tregs, must ensure stability, as well yielding a safe and efficacious product whether the product is fresh or frozen (Capelli et al., 2022).

### 3.4 Release criteria

The interrogation of the final product and through regular sampling during the manufacturing process benefits the overall safety and functionality of Tregs, i.e., efficacy of the therapeutic agent. All *ex-vivo* cell expansion protocols are associated with the risk of microbiological contaminations and accumulation of aberrations. Therefore, the manufacturing process and the decision for release of the final product must include and consider checks and controls to mitigate the risks. These include product-related parameters such as identity, purity and functionality, including the classical safety parameters such as sterility, endotoxin content, absence of *mycoplasma* and proof of removal of any expansion beads used. The quality control assessments include performing karyotyping to screen for chromosomal aberrations. More specifically, for Tregs, it is essential that GMP manufacturing includes regular checks and controls safeguarding their suppressive activity by regular monitoring of FOXP3 levels and thereby ensuring the final therapeutic product can be safely administered. These parameters, as well as the final product’s phenotypic characteristics, are included in cell product release criteria. The parameters tested to assess the product will vary depending on the type of product and the study.

In addition to release relevant criteria, additional data are gathered for better understanding and characterization of the Treg products during clinical trials. Regular measurement of Treg-specific demethylated regions (TSDR), upstream of exon 1 of FOXP3 gene, is desirable, as in effector T cells this region is completely methylated while for stable suppressive Tregs it is constitutively demethylated. Therefore, functional tests with autologous responders in the presence of expanded Tregs, i.e. proliferation of effector T cell suppression assay or MLR suppression assays, are considered as the gold standard of quality checks for expanded Tregs (Bluestone et al., 2015; Brunstein et al., 2016; Wiesinger et al., 2017; Mathew et al., 2018; Roemhild et al., 2020). However, the assays designed to measure Treg function are complex and challenging, which may compromise validity and precision of the data obtained. There are numerous advanced ‘omic’ technologies now available which permit detailed analysis of the cellular products. These can assess in considerable detail the biological systems; clonality, methylation (epigenetics) and overall functionality of the living drug products (McNamara et al., 2015).

### 3.5 Optimising treg functions

The potency of Treg products may be enhanced by considering the antigen-specificity of Tregs (Landwehr-Kenzel et al., 2014). Strategies to expand donor-specific Tregs in a GMP-compliant manner out of a mixed lymphocyte reaction (MLR) using belatacept (Nulojix®), which inhibits co-stimulatory signals have been reported (Guinan et al., 2016). This strategy reportedly leads to Treg products with a high TSDR demethylation and absence of effector T cell characteristics upon donor-specific re-stimulation (Guinan et al., 2016). Equally, co-culture with allogenic dendritic cells (Yamazaki et al., 2006; Kasahara et al., 2017; Alvarez-Salazar et al., 2020) or B cells (Levitsky et al., 2011; Landwehr-Kenzel et al., 2014) is employed to obtain alloantigen-specific Tregs for clinical applications in transplantation.

More recently, the advances in genome editing, in particular, clustered regularly interspaced short palindromic repeat/CRISPR associated protein 9 (CRISPR/Cas 9), to generate antigen-specific Tregs and/or enhance the expression of Treg specific genes have increased the therapeutic potential of ATMPs (NCT04817774). This technology provides means with which to alter gene expression profile with the aim of conferring a competitive survival advantage to Tregs (Amini et al., 2021). For example, prospective recipients of Treg products are commonly managed with immunosuppressive drugs, which can interfere with Treg functions (San Segundo et al., 2006; Presser et al., 2009), therefore CRISPR gene editing could be used to engineer Treg products which are resistant to the IS pharmaceuticals such as calcineurin inhibitors (Amini et al., 2020; Peter et al., 2022).

## 4 Clinical trials of adoptive regulatory T cell therapy

The potential of adoptive Treg therapy in the clinical management of diverse immunopathologies is illustrated by the multitude of clinical trials registered (Supplementary Table S1). While adoptive Treg therapy is predominantly used in the management of SOT and HSCT, it is also increasingly used to treat autoimmune diseases, (neuro-)degenerative diseases such as liver cirrhosis, Amyotrophic Lateral Sclerosis or Alzheimer’s disease (Supplementary Table S1). However, there is no consensus on the optimum conditions developed to generate Tregs which is also reflected in the diversity among the protocols described (Supplementary Table S1). This makes inter-data comparison difficult. In addition to the single use of Treg products, they have also been used in concomitant therapies, which include a range of drugs/strategies as well as other cell products (mesenchymal stem cells or respective ratios with donor effector T cells as replacement for unclassified donor-lymphocyte infusions neglecting regulatory or effector classification) (Supplementary Table S1). Both polyclonal and antigen specific Tregs are being tested. A number of centres use pre-conditioning similar to applications of effector T cells (Brunstein et al., 2011).

### 4.1 Solid organ transplantation

Among the SOT recipients Treg therapy has most commonly been used to manage kidney transplant recipients, which is by far the most common organ transplant performed. The pioneering studies described managing subclinical inflammation in kidney transplants with Treg therapy. One such report included treating three recipients with approximately 3.2 × 10^6^ polyclonal Treg, 6 months post-transplant. Apart from neutropenia the three patients experienced no other adverse events (Chandran et al., 2017). By infusing deuterium labelled Tregs, the investigators were able to monitor their distribution in the recipients’ circulation for up to a month. Two of the subjects showed decreased numbers in lymphocytes associated with inflammation in their follow-up biopsies. In the third patient diagnosed with *de novo* donor-specific antibody formation, the data indicated no noticeable of improvement in inflammatory status (Chandran et al., 2017). The starting material was collected prior to the transplantation, however, collecting 400 ml of blood from frail end stage renal disease anaemic patients may be problematic. Data from 9 kidney transplant recipients show infusion of large Treg doses of up to .5 to 5.0 billion 60 days post-transplant switch from calcineurin to mTOR inhibition. This approach yielded an efficient and sustainable increase in peripheral blood Treg numbers for up to 1 year without adverse events (Mathew et al., 2018). The strategy of combining Treg infusion with mTOR inhibition has now also been successfully applied by other researchers (Gedaly et al., 2019). With the end point including safety and efficacy, ‘The ONE study’ was a first in human multicentre cellular therapy clinical trial involving researchers in Europe and the United States. This trial was also able to consider standardization and harmonization relating to manufacturing standard operating procedures, dosage, and standard of care procedures with view to optimizing clinical management of kidney transplants (Sawitzki et al., 2020). As part of the ONE study international consortium, our centre conducted a clinical trial (ONEnTReg13), in which polyclonal Treg products were administered to living donor kidney recipients 14 days post-transplantation. The trial revealed no toxicities or increases in infectious complications (Roemhild et al., 2020). This seminal study demonstrated the safety and tolerability of *ex-vivo* expanded autologous Treg products in managing the vast majority of the treated renal transplant recipients (Roemhild et al., 2020) and significantly, the study provided promising preliminary efficacy data: Treg infusions permitted tapering the triple agent based immunosuppressive therapy to low dose Tacrolimus monotherapy in 73% of the patients with a concurrent reduction in activated effector T cells (Roemhild et al., 2020). These clinical data are supported by T cell receptor β-chain sequencing analysis of recipients’ blood samples which indicated a preferential expansion of graft-specific Treg products (Roemhild et al., 2020). However, a stable increase in the frequencies of Tregs in circulation could not be observed. This may be due to the tissue homing and the comparatively low doses of .5–3 million Tregs/kg (Roemhild et al., 2020). Building on the encouraging findings from the ONEnTreg13 study, the possibility of extending this approach to deceased kidney transplants is being explored, given the significantly greater number of these recipients (ProTreg study—in preparation). A United Kingdom centre included in ‘The ONE study’ reported improved rejection-free survival of Treg-treated kidney transplant recipients at 48 months post-transplant without safety concerns and 4 patients on Tacrolimus monotherapy (Harden et al., 2021).

The utility of Treg therapy has also been trialled among liver transplant patients. Interestingly, the observations of spontaneous graft tolerance increasing with time from the liver transplant, ranging from 10% in 2–6 years post-transplant to >40% after 10 years, might provide opportunities for more ambitious protocols in hepatic transplantation. In 2020, Sanchez-Fueyo *et al.* reported a FIH Phase I/IIa clinical trial (NCT02166177) with 9 liver transplant recipients which investigated the safety and biological activity of adoptively transferred autologous Tregs (Sanchez-Fueyo et al., 2020). The study confirmed the safety of the adoptive Treg therapy, with only one reported infusion reaction, which was attributed to dose limiting toxicity (Sanchez-Fueyo et al., 2020).

### 4.2 Haematopoietic stem cell transplantation

Allogeneic HSCT is a curative therapy option for diverse cancers, however, genetic differences between donor and host can lead to GvHD, a major cause of morbidity and mortality. Currently a number of clinical trials involving infusion of donor-derived Tregs post HSCT patients are on-going (Supplementary Table S1). However, one such trial has reported infection complications in all trial subjects, despite the relatively low Treg doses infused (Supplementary Table S1). In contrast to SOT, the management of HSCT patients often relies entirely on Tregs with subsequent donor derived conventional T cell infusions, *i.e*. donor lymphocyte infusions (DLI) to manage the underlying disease (Di Ianni et al., 2011). In Di Ianni *et al.*, studied 28 patients, who received adoptive transfer of Tregs, no GvHD post HSCT in absence of IS therapy was reported (Di Ianni et al., 2011). The investigators also observed improved lymphoid reconstitution with decreased infection complications, without compromising the graft *versus* leukaemia (GVL) effect (Di Ianni et al., 2011) (Martelli et al., 2014). These data were supported by another study which reported finding 75% of the subjects experienced a marked reversal in their GvHD symptoms and achieved leukaemia free survival, despite the absence of IS agents (Haematologica, 2020). In deviation from other clinical trials, these studies infused non-cryopreserved Treg products. A comparable trial compared fresh and cryopreserved Tregs and found that the efficacy of cryopreserved Tregs was markedly inferior (Meyer et al., 2019). The cells were sorted by flow cytometry and activated using magnetic beads. Researchers have reported chronic and acute GvHD case studies; the subject with chronic GvHD benefitted from adoptive transfer of FACS-isolated Tregs and the IS medication could be dramatically reduced, while the other individual with acute GvHD experienced transient improvement in symptoms, but subsequently died due to multiorgan dysfunction (Trzonkowski et al., 2009). Another trial adoptively transferring Tregs and DLI after haploidentical transplantation for treatment of acute myeloid leukaemia showed low leukaemia relapse and GvHD rates (Pierini et al., 2021).

A combined Phase I/IIa trial investigated the adoptive transfer of very low numbers (500,000) of freshly isolated Tregs into high GvHD risk HSCT recipients previously suffering from leukaemia followed by infusion of effector T cells 8 weeks later without observing toxicities, acute GvHD or infections (Edinger and Hoffmann, 2011).

Based on the notion that CD45RA identifies Tregs with greater stability (Arroyo Hornero et al., 2017), it has been used to select for this subset of Tregs when treating steroid-resistant severe acute gastrointestinal GvHD patients. Reports suggest it leads to an increase in Treg numbers in patients (Edinger, 2016).

Trials in which third party Treg products were infused have also been performed following umbilical cord blood transplantation (Brunstein et al., 2011). The studies concluded good overall tolerance with concurrent reduction in the risk of GvHD development and without noticeable adverse events (Brunstein et al., 2011). Investigators have also explored infusing expanded alloantigen-specific Tregs derived from co-cultures with recipient dendritic cells to eliminate the risk of GvHD post HSCT (Supplementary Table S1). Overall HSCT patients experienced an improved clinical outlook due to the marked alleviation in the GvHD symptoms. The investigators have reported improvements in lymphoid reconstitution and decreased infectious complications. However, a major concern with successful GvHD therapy using Tregs has been that cellular therapy would result in global IS and so interfere with effective GVL response in haemato-oncologic indications. While pre-clinical trials do not support this as a risk, clinical trials data for cancer recurrence are not sufficiently mature to reach a conclusion.

The encouraging data from adult living donor kidney transplant recipients at our institute have led to a cautious extension of similar trials to infusion of polyclonal Tregs to paediatric HSCT recipients. The recent pilot trial at our institute investigated the similar Treg product for treatment of paediatric chronic GvHD on compassionate basis (Landwehr-Kenzel et al., 2022). All treated patients experienced major clinical improvement, although one patient, who was suffering from aspergillosis, experienced only transient improvements and subsequently died (Landwehr-Kenzel et al., 2022).

### 4.3 Autoimmunity and beyond

The therapeutic potential of Tregs is now increasingly exploited to ameliorate deleterious effects of more autoimmune and neurodegenerative disease (Supplementary Table S1). For example, clinical management of amyotrophic lateral sclerosis or Alzheimer’s disease. Similarly treating liver cirrhosis patients with Tregs is being explored. The promise of cellular therapies in such diverse immune disorders will provide opportunities for unmet medical needs.

Since the developments in *ex-vivo* expansion of Tregs, investigators have explored the utility of adoptive Treg therapy to treat autoimmune disorders, such as systemic lupus erythematosus (SLE), including type 1 diabetes (Supplementary Table S1).

The therapeutic potential of Tregs has extended to trials involving the rapidly developing field of islet xenotransplants to treat diabetes mellitus (Supplementary Table S1). Bluestone *et al.*, explored the possibility of treating newly diagnosed type 1 diabetes (T1D) patients with Treg adoptive immunotherapy (Bluestone et al., 2015; Gitelman and Bluestone, 2016). They enrolled 16 T1D patients for an open label interventional Phase 1 trial with the objective of assessing the safety and feasibility of intravenous infusion of *ex-vivo* expanded autologous polyclonal Tregs (Bluestone et al., 2015; Gitelman and Bluestone, 2016). After a mean follow-up period of 31 months, only three severe adverse events were reported, and no opportunistic infections were reported (Bluestone et al., 2015; Gitelman and Bluestone, 2016). But the investigators concluded none of the observed adverse events, ranging from mild to severe, were related to the cell infusions. These findings confirmed the proof of concept such the feasibility of treating T1D with suppressive Tregs and that the approach was concluded to be safe. However, the investigators were unable to conclude regarding the optimal Treg dose and whether the Tregs had an effect on the islet cells. Therefore, a multi-centre Phase II randomized blind study (NCT02691247) was subsequently established to address these issues. The data are not currently available. In addition, there are number of T1D studies on-going, as listed in Supplementary Table S1, involving Treg infusion, some of which even reported cases of paediatric patients becoming independent of insulin after Treg infusion (Marek-Trzonkowska et al., 2012).

Desreumaux *et al.* reported the first-in-human phase I/IIa open label study involving 20 patients with active and symptomatic refractory Crohn’s disease receiving ovalbumin-specific Tregs (Desreumaux et al., 2012). Despite the severe adverse events, which the researchers reported were related to the natural history of the disease, they concluded the cell infusions to be safe and reported dose-dependent efficacy (Desreumaux et al., 2012). The utility of polyclonal Tregs to treat this disease is currently being assessed (Goldberg et al., 2019).

To investigate, whether infusion of autologous Tregs in patients with amyotrophic lateral sclerosis (ALS) is safe and tolerable, Thonhoff and colleagues (Alsuliman et al., 2016; Thonhoff et al., 2018) treated three ALS patients with *ex-vivo* expanded polyclonal Tregs at a dose of 1 x 10^6^ cells/kg per infusion. Each patient received 8 doses in total, four doses at early stages (every other week over 2 months) and four doses at later stages (every 4 weeks over 4 months) of disease. Infusions of Tregs were safe and well tolerated in all patients and slowed the progression rates during early and later stages of disease, as assessed by the Appel ALS score. The slowing of disease progression correlated with Treg suppressive function.

Dall’Era and colleagues assessed the efficacy of autologous Tregs in patients with cutaneous manifestations of SLE and tissue inflammation (Dall'Era et al., 2019). However, the study was prematurely terminated with only one patient recruited, who received one infusion of autologous, deuterium labelled polyclonal Tregs (100 x 10^6^ cells). At this dose, there was no demonstrable clinical impact. But the investigators did highlight that despite having administered strong immunosuppressive triple therapy for years, (hydroxychloroquine, MMF, and prednisone) it was possible to isolate and expand Treg cells from the patient.

Many more approaches are currently ongoing and results are awaited (Supplementary Table S1).

## 5 Conclusion

Tregs have very rapidly emerged as critical to immune homeostasis and the ongoing early phase clinical trials imply, we are at the cusp of cellular therapy revolution in managing auto- and alloimmunity. The reported data provide evidence-based opportunities to embark on next wave of adoptive Treg therapy innovations. Gene editing technologies herald opportunities to improve antigen specificity, stability and function of therapeutic Tregs, with the aim of increasing fitness to tailored specificities (Amini et al., 2021; Arjomandnejad et al., 2022) and there are clinical trials in the pipeline (NCT04817774). However, a number of questions remain, for example, exactly which antigens drive proliferation and regulate their function. Equally, the precise mechanisms which modulate the immune system to promote tolerance remains ambiguous. Non-etheless, adoptive Treg therapy is now widely accepted as a promising therapeutic tool. The clinical trials have also highlighted difficulties which must be addressed; finding optimal dosing and time points for application as well as the ideal combinatorial treatments. The investigators must also address the challenges related to GMP compliant manufacturing of these living drugs and the associated costs. Some limitations may be overcome with the rapidly increasing gene engineering technologies, which can elegantly optimize the Treg properties for the respective application, e.g., by redirecting the specificity, increasing the fitness or other functional properties, which are extensively reviewed elsewhere (Amini et al., 2021). Furthermore, most of the early-stage manufacturing processes rely on individual product manufacture for each patient as required, autologous for SOT or donor-derived for HSCT. The possibility of using third party-derived products, currently being investigated, should if successful, address the concerns of broad supply and reduction in treatment costs and the considerable expectation for the first effector T cell-based approved advanced therapeutic medicinal products. To date clinical trials have shown enormous promise in Treg therapies. The era of adoptive Treg therapy is on the horizon, with advances in our understanding of the mechanisms will enable us to exploit the novel therapeutics.
