# Supplementary material for: Clinical adoptive regulatory T Cell therapy: State of the art, challenges, and prospective
Source: Front Cell Dev Biol. 2023 Jan 30;10:1081644. doi: 10.3389/fcell.2022.1081644 (PMC9924129; doi:10.3389/fcell.2022.1081644)
Supplement: Supplementary file 1 [file Table1.DOCX]

**Table 1. Clinical Trials.** List of studies investigating the clinical application of Tregs (Interventional trials). (data cut-off: 2022/10/17)

| **Indication** | **Products/application** | **Registry ID / Reference (x)** | **Key information** |
| --- | --- | --- | --- |
| **SOT** | | | |
| Late subclinical inflammation in kidney transplantation | Peripheral blood (400 ml)-derived 320 million polyclonal autologous CD4^+^ CD127^low/-^ CD25^+^ cytometry-sorted Treg on the day of the 6 month surveillance biopsy | NCT02088931  (Chandran et al., 2017) | Phase I  Enrolment (actual): 3 participants  Recruitment status: Completed  safe and well tolerated |
| Subclinical inflammation in kidney transplantation | 550 +/- 450 million polyclonal Tregs (at least 300 million Tregs) infused on day of surveillance biopsy (6 months post-transplant) | NCT02711826 | Phase I/II  Enrolment (estimated): 14 participants  Recruitment status: recruiting |
| Living-donor kidney transplant recipients | 0.5/1/2.5-3 million/kg polyclonal autologous Tregs derived from 40-50ml peripheral blood infused on d7 after transplantation | NCT02371434  (Roemhild et al., 2020; Sawitzki et al., 2020) | Phase I/II  Enrolment (actual): 17 patients  Recruitment status: Completed  Safety and tolerability for at least 3 years after single infusion, reduction of triple drug immunosuppression to monotherapy with Tacrolimus while the reference groups stayed on double or triple immunosuppressive therapy |
| Living donor kidney transplantation without need for dialysis in the 1st week after transplantation | Induction therapy with Alemtuzumab (one month post-transplant), leukapheresis (frozen)-derived 0.5/1/5x10^9^ polyclonal autologous CliniMACS CD4/CD25-enriched Tregs, infused on day 60 after transplantation (while on mTOR inhibitor + Mycophenolic Acid) | NCT02145325  (Mathew et al., 2018); Acronym: TRACT trial | Phase I/II  Enrolment (actual): 10 patients  Recruitment status: Completed  5–20-fold increase in the percentages of Tregs in all subjects stable at least until one year after infusion |
| Kidney transplant recipients using Everolimus | Leukapheresis-derived intravenous CliniMACS CD25-enriched Tregs | NCT03284242;  (Gedaly et al., 2019) | Phase I/IIb  Enrolment (estimated): 12 participants  Recruitment status: recruiting |
| Matched living donor kidney transplantation | 1 x 10^4^ to 2 x 10^6^ cells/kg from *ex-vivo* mixed lymphocyte reaction (donor/recipient) co-culture with belatacept expanded Tregs transferred at d7 after transplantation | NCT02091232;  (Guinan et al., 2016) | Phase I  Recruitment (actual): 5 participants  Recruitment status: completed  No results posted (clinicaltrials.gov registry); see published references |
| HLA-mismatched living donor kidney transplantation | ATG and irradiation 2 weeks prior to 25 million/kg of recipient Tregs in combination with 100 million/kg of donor hematopoietic stem cells | NCT03943238 | Phase I  Enrolment (estimated): 22 participants  Recruitment status: recruiting |
| Living donor kidney transplantation | 1-10 million/kg of Tregs infused 5 days after transplantation | NCT02129881;  (Fraser et al., 2018; Harden et al., 2021) | Phase I/II  Treg Therapy cohort: 12 participants  Reference cohort: 19 participants  no toxicities after infusion/ no excess safety concerns  UK arm of ‘The ONE Study’ (The ONE Study UK Treg Trial)  No results posted (clinicaltrials.gov registry); see published references |
| Living donor kidney transplantation | Recipient Tregs combined with donor bone marrow | NCT03867617 | Phase I/IIb (safety/efficacy)  Enrolment (estimated): 12 participants  Recruitment status: recruiting |
| De novo living donor kidney transplantation | 300/900 million autologous alloantigen-reactive Tregs | NCT02244801 | Phase I/II  Enrolment (actual): 6 participants  Recruitment status: completed  No results posted yet (clinicaltrials.gov registry) |
| Paediatric kidney transplantation | Alemtuzumab 3 and 2 weeks before transplantation and at the day of transplantation, frozen autologous CD4+ cells (from 2 x 70ml PB)-derived subcutaneous 200 million Tregs a month and 180 days after transplantation | NCT01446484 | Phase I/II  Enrolment (estimated): 30 participants  Recruitment status: unknown |
| HLA-A*02 positive mismatched living donor kidney transplantation | Autologous expanded CD4+/ CD45RA+/ CD25+/ CD127_low_ - retroviral transduced anti-HLA-A*02 CAR-Tregs infused 12 weeks after transplantation | NCT04817774; EudraCT 2019-001730-34 | Phase I/II  Enrolment (estimated): 42 participants  Recruitment status: recruiting |
| Kidney and liver transplantation | Autologous CD4+ CD25+ CD127low CD45RA+ naïve Tregs derived from leukapheresis, amplified *ex-vivo* for 10 days,  Preclinical validation of Treg batches (PRE-TREG) | NCT04661254 | Phase: NA  Enrolment (estimated): 4 participants  Recruitment status: Not yet recruiting |
| Liver transplantation | Peripheral blood or leukapheresis-derived 50/200/800 million autologous donor alloantigen-reactive Tregs (exposed to donor liver cells during expansion) | NCT02188719 | Phase I  Enrolment (actual): 15 participants  Recruitment status: Terminated due to (a) high number of ineligible subjects, (b) slow enrolment & (c) manufacturing difficulties within the constraints of the funding period |
| Liver transplantation | Peripheral blood (150ml)-derived autologous CliniMACS enriched cryopreserved 1/ 4.5 million Tregs administered 3 months post-transplant | NCT02166177;  (Safinia et al., 2016; Sánchez-Fueyo et al., 2020; Whitehouse et al., 2015) | Phase I/II  Enrolment (actual): 9 participants  Recruitment status: completed  No results posted (clinicaltrials.gov registry); see published references |
| Chronic rejection of liver transplants (1 – 10 years post-transplant) | Peripheral blood-derived multiple infusions of 1 million/kg of autologous CD4+ CD25+ CD127- donor alloantigen-expanded Tregs | NCT01624077 | Phase I/II  Enrolment (estimated): 1 participant  Recruitment status: unknown |
| Liver transplantation | Leukapheresis or peripheral blood-derived donor-reactive intravenous 400 million Tregs | NCT02474199 | Phase I/II  Enrolment (actual): 15 participants (Treg therapy + IS withdrawal arm: 5/15 participants)  Recruitment status: completed  Results (Treg therapy + IS withdrawal arm):  All-cause mortality: 0/5 (0 %), SAEs (Transplant rejection): 80% (4/5) in Treg receiving group vs. 20% (1/5) in control group |
| Liver transplantation | Leukapheresis-derived 2.5 million autologous donor alloantigen-reactive CD4+ CD127_low_ /- CD25+ Tregs | NCT03577431 | Phase I/II  Enrolment (estimated): 9 participants  Recruitment status: recruiting |
| Living donor liver transplantation | 2-week co-culture of recipient leukapheresis-derived lymphocytes + irradiated donor lymphocytes + anti-CD80/86-derived 0.23 - 6.37 million induced Tregs/kg | (Todo et al., 2016) | Pilot study: Seven (non-autoimmune) patients completely free of immunosuppressive drugs, Three patients (autoimmune liver failure) on low dose immunosuppression due to mild acute cellular rejection symptoms upon weaning |
| Living donor liver transplantation | 2-week co-culture of recipient leukapheresis-derived lymphocytes + irradiated donor lymphocytes + anti-CD80/86-derived induced Tregs | NCT04950842 | Phase I/II  Enrolment (estimated): 10 participants  Recruitment status: recruiting |
| Islet transplantation | Autologous peripheral blood (400 ml)-derived 4 - 16 x 10^8^ polyclonal CD4+ CD127_low_ /- CD25+ Tregs 6 weeks post-transplant. | NCT03444064 | Phase I/II  Enrolment (estimated): 18 participants  Recruitment status: Active, not recruiting |
| Islet transplantation | Autologous leukapheresis-derived sorted frozen Tregs infused in parallel with the transplantation | NCT04820270 | Phase I/II  Enrolment (estimated): 8 participants  Recruitment status: Active, not recruiting |
| Islet xenotransplantation | 2 million/kg of autologous Tregs | NCT03162237 | Phase I/II  Enrolment (actual): 20 participants  Recruitment status: completed  No results posted yet (clinicaltrials.gov registry) |
| Paediatric heart transplantation | 10/20 million autologous thymic tissue-derived Tregs/kg expanded for 7 – 10 days | NCT04924491  (de Quirós et al., 2018) | Phase I/II  Enrolment (estimated): 11 participants  Recruitment status: Recruiting |
| Renal transplant recipients | Obtain naturally occurring regulatory T cells (Treg) from patients prior to transplantation and re-infuse after expansion into the transplant recipient 6 months post-transplantation | EudraCT:  2017-001421-41 | Phase IIb trial, single centre, open-label, randomised-controlled  Aim for recruitment: 68 patients over 3 years |
| Liver transplant recipients | Exploring cellular therapy to facilitate immunosuppression withdrawal in liver transplant recipients; Target dose is at least 90 x 10^6 total cells; Single dosing. | NCT03654040 | Phase I/II  Enrolment (estimated): 9 participants  Recruitment status: recruiting |
| **GvHD/HSCT** | | | |
| Double umbilical cord blood transplantation | Cyclophosphamide + fludarabine preconditioning, pre-treatment with acetaminophen and diphenhydramine, 0.1-30 × 10^5^ umbilical cord (3rd party with 4-6/6 HLA matching)-derived Tregs/kg on the day after umbilical cord blood transplantation once or twice (on day 15) | NCT00602693  (Brunstein et al., 2011) | Phase I  Enrolment (actual): 41 participants  Recruitment status: completed  First in human clinical trial: no toxicities, Tregs detectable in peripheral blood for 14 days, in comparison to historical control reduced incidence of grade III/IV GvHD |
| Non-myeloablative umbilical cord blood transplant (haematological malignancy) | 1 – 24 h after 2nd cord blood infusion polyclonal Tregs | NCT02991898 | Phase II  Enrolment (actual): 3 participants  Recruitment status: terminated due to considering new technology for product |
| Steroid-refractory chronic GvHD | 0.5/1/2-3 million/kg of donor-derived Tregs (CliniMACS CD19/CD8-depleted; CD25 enriched) | NCT02385019 | Phase I/II  Enrolment (estimated). 22 participants  Recruitment status: unknown |
| Haploidentical HSCT | Donor-derived 0.1/0.3/ 1/3 million/kg Tregs in combination with Tconv cells | NCT01050764 | Phase I/II  Enrolment (actual): 10 participants  Recruitment status: Terminated due to safety issues (infections in all participants) |
| Haploidentical HSCT | Fresh leukapheresis-derived 2/4 million/kg of donor-derived CliniMACS CD8/CD19-depleted CD25-enriched Tregs (4 days prior to transplantation) combined with Tconv infusion | (Di Ianni et al., 2011) | Phase I/II  Enrolment (actual): 28 participants  Recruitment status: completed  Results: prevention of GvHD without any post-transplant immunosuppression, improved lympho-reconstitution, improved anti-pathogen immunity, maintenance of graft-versus-leukaemia effect |
| Haploidentical HSCT | Fresh leukapheresis-derived 2/4 millions/kg of donor-derived CliniMACS CD8/CD19-depleted CD25-enriched Tregs (4 days prior to transplantation) combined with effector T cell infusion | (Martelli et al., 2014) | Phase II  Enrolment (actual): 43 participants  Recruitment status: completed  Results: protection from GvHD without any post-transplant immunosuppression, maintenance of graft-versus-leukaemia effect |
| Allogenic T cell depleted HSCT | Fresh leukapheresis-derived 2 million/kg of donor-derived CliniMACS CD8/CD19-depleted CD25-enriched Tregs (4 days prior to transplantation) combined with effector T cell infusion | NCT03977103; (Pierini et al., 2021) | Phase II  Enrolment (estimated): 80 participants  Recruitment status: recruiting  Without any post-transplant immunosuppression, 75% GvHD-free and relapse-free survival at 29 months follow up |
| Severe steroid-refractory chronic GvHD | 3x intravenous 0.017/ 0.033/ 0.066 million/kg of donor leukapheresis-derived CliniMACS CD19/CD8-depleted CD25-enriched Treg infusions | NCT02749084 | Phase I/II  Enrolment (estimated): 20 participants  Recruitment status: recruiting |
| HLA-matched HSCT | Donor GSCF mobilized peripheral blood-derived 1/3 million/kg of CliniMACS CD25-enriched flow cytometry-sorted CD4+ CD127_low_ CD25+ Treg infused on the day of transplantation (5 frozen, 7 fresh products) | NCT01660607  (Meyer et al., 2019) | Phase I/II  Enrolment (actual): 68 participants  Recruitment status: Active, not recruiting  Preliminary results: Safety & tolerability, no GvHD in patients infused with fresh Tregs, but 2/5 patients infused with cryopreserved Tregs developed GvHD $\geq$grade II |
| Steroid-refractory chronic GvHD | One infusion of ≥0.5 x10^6^ Tregs/kg at 60-90 days after first day of rapamycin administration and after CNI discontinuation. Plus IL-2 1x10^6^ IU/day on day of Treg infusion for a period of 2 months. | NCT01903473 | Phase II  Enrolment (actual): 19 participants  Recruitment status: Terminated due to slow recruitment |
| Steroid-refractory chronic GvHD | Intravenous Treg-enriched donor lymphocytes combined with daily IL-2 infusion (8 weeks) | NCT01937468 | Phase I  Enrolment (actual): 25 participants  Recruitment status: Active, not recruiting |
| HLA identical sibling HSCT | Donor-derived alloantigen-specific Tregs derived from co-culture of recipient dendritic cells, infused 2 days prior transplantation | NCT01795573 | Phase I  Enrolment (actual): 38 participants  Recruitment status: Active, not recruiting |
| Non-myeloablative sibling peripheral HSCT | 3/30/300/1000 millions/kg of iTregs > 4 h prior to transplantation; single centre dose escalation | NCT01634217 | Phase I  Enrolment (actual): 16 participants  Recruitment status: completed  No results posted yet (clinicaltrials.gov registry) |
| Myeloablative HSCT | Donor-derived Tregs and conventional T cells | NCT03977103 | Phase II:  Recruitment status: recruiting |
| myeloablative allogeneic HSCT; hematologic malignancies | Tregs combined with conventional T cells; T cell-depleted graft  Product acronym TregGraft: Orca-T | NCT04013685 | Phase Ib, multicentre  Enrolment (estimated): 84 participants  Recruitment status: recruiting |
| HSCT for treatment of ß‑thalassemia | Donor-derived Treg infusion 60 days after transplantation | NCT03101423 | Phase: NA  Enrolment (estimated): 30 participants  Recruitment status: unknown |
| HSCT (high risk of leukaemia – prevention of GvHD) | up to 50 x 10⁵ freshly isolated Tregs from stem cell donor within 1 year after transplantation, 8 weeks later, equal cell number of Tconv | (Edinger & Hoffmann, 2011) | Phase I/II  Enrolment (actual): 9 participants  Recruitment status: completed  no toxicities, no acute GvHD, no increased infection rate |
| allogenic HSCT (Treatment of chronic GvHD, Grade IV acute GvHD) | 1 x 10⁵ (chronic GvHD) or 30 x 10⁵ (acute GvHD, three infusions) polyclonal *ex-vivo* expanded donor-derived CD4+CD25+CD127- Tregs isolated by FACS after magnetic CD4 enrichment | (Trzonkowski et al., 2009) | Case Reports (Two patients): no toxicities  •Chronic GvHD: withdrawal of MMF, reduction of steroids, significant improvement in the bronchial obturation cessation of bronchodilators, increase in blood Tregs (6 months after transfer)/ decrease in serum levels of cytokines  •Acute GvHD: transient improvement in symptoms, but patient died from multiorgan dysfunction |
| Steroid-resistant severe acute gastrointestinal GvHD | CD45RA^+^ 1 – 3 million donor-derived expanded Tregs/ml | EudraCT 2012-002685-12;  (Edinger, 2016) | Increase in Treg pool of several patients |
| steroid-resistant chronic GvHD | 3 – 10 million donor-derived expanded Tregs/g | EudraCT 2016-003947-12 |  |
| steroid-refractory chronic GvHD | Donor-derived magnetically isolated Tregs + Rapamycin | EudraCT 2012-000301-71 |  |
| steroid dependent/ refractory chronic graft versus host disease | Donor regulatory T cells: 1x10^5^ Tregs/kg (cohort 1), 5x10^5^ Tregs/kg (cohort 2), 1.5x10^6^ Tregs/kg (cohort 3) with an extension phase at the MTD (or maximum administered dose if the MTD is not reached) | NCT01911039 | Phase I (safety/ tolerability)  14 participants  Recruitment status: completed  No results posted yet (clinicaltrials.gov registry) |
| steroid-refractory chronic GvHD with no complete remission under ruxolitinib | Dose escalation sequential cohorts Regulatory T-cell enriched infusion (cells/kg): 0.5 x 10^6^ cells/kg (Dose level A), 1 x 10^6^ cells/kg (Dose level B), 2 x 10^6^ cells/kg (Dose level C) | NCT03683498 | Phase I (safety and maximum tolerated dose-level)  16 participants  Recruitment status: completed  No results posted yet (clinicaltrials.gov registry) |
| GvHD affecting the liver or gastrointestinal organs (visceral) within 100 days (acute) after undergoing a hematopoietic stem cell transplant | Donor regulatory T lymphocytes IV over 5 minutes or less on day 0. Some patients receive second infusion of frozen donor Tregs 5-7 days after the initial infusion or 2 additional infusions separated by 5-7 days.  *Comment:* No specific dose communicated (clinicaltrials.gov) | NCT02526329 | Phase I  Enrolment (estimated): 12 participants  Recruitment status: suspended (logistics) |
| steroid-refractory chronic graft versus host disease with no complete remission under ruxolitinib | Enrichment of CD25hi Tregs from CD8 and/or CD19 pre-depleted leukapheresis products  Doses of Treg cell enriched infusion will be 2x10^6 cells/kg | NCT05095649 | Phase II  Enrolment (estimated): 15 participants  Recruitment status: recruiting |
| steroid-refractory or dependent chronic graft vs host disease | Continuous Alloreactive T Cell Depletion and Regulatory T Cell Expansion; Autologous peripheral blood mononuclear cells ex vivo depleted for reactive T cells, using TH9402 based photodynamic therapy, in a final formulation of 10% DMSO, 30% autologous plasma in PlasmaLyte | NCT02519816 | Phase II multicentre Canadian only study  Enrolment (actual): 17 participants  Recruitment status: active, not recruiting |
| patients who will receive a MRD, a MUD, or cord blood transplant;  prevent or reduce the effects of GvHD | Experimental Phase I: Fucosylated Tregs (1 x 10^6/kg) + Chemotherapy vs. Non-fucosylated Tregs (1 x 10^7/kg) + Chemotherapy  Experimental: Phase II: Fucosylated Tregs (1 x 10^7/kg) + Chemotherapy vs. Non-fucosylated Tregs (1 x 10^7/kg) + Chemotherapy | NCT02423915 | Phase I  Enrolment (actual): 5 participants  Recruitment status: completed  No results posted yet (clinicaltrials.gov registry) |
| relapsed/refractory AML and/or myelodysplastic syndromes (MDS) receiving a haploidentical donor allogeneic HSCT | haplo Treg-enriched donor cells  Given intravenously -4 day prior to HSCT  *Comment:* No specific dose communicated (clinicaltrials.gov) | NCT04678401 | Phase I  Enrolment (estimated): 10 participants  Recruitment status: recruiting |
| grade II-IV acute graft-versus-host disease (GvHD) in recipients of non-myeloablative double UCB transplantation | fixed dose ratio to the combined CD3+ cell count;  Treg:CD3+ cells ratio of 5:1;  nTreg cell dose depends on the CD3+ cell content of the two graft UCB graft units | NCT02118311 | Simon's optimal two-stage phase II trial  Enrolment (actual): 0 participants  Recruitment status: withdrawn (changing study design; will replace with a different protocol) |
| High-risk leukemia or other hematologic diseases (Lymphoma, Multiple Myeloma & Plasma cell neoplasm, Myelodysplastic Syndromes, Secondary Myelofibrosis | On day -1 prior to UCB transplantation, Treg cells will be infused IV without in-line filtration.  Semi-log dose escalation of CD4+CD25+ Treg cells;  Dose: 0.1 x 10^6/kg, 0.3 x 10^6/kg, 1 x 10^6/kg and  3 x 10^6/kg weight | NCT00376519 | Phase I  Enrolment (actual): 3 participants  Recruitment status: terminated (slow accrual) |
| Patients with advanced hematologic malignancies undergoing reduced intensity allogeneic HCT | Purified Tregs plus CD34+ HSPC;  Dose: 1x10^6 cells/kg to 3x10^6 cells/kg | NCT05088356 | Phase I  Enrolment (estimated): 24 participants  Recruitment status: recruiting |
| Patients with high-risk hematologic cancer who are undergoing donor peripheral blood stem cell transplant | CD4+/CD25+ Tregs given IV over 15-60 minutes on Day -2 (prior to peripheral blood progenitor cell transplant);  Cohort 1: 3x10^6 Tregs/kg; Cohort 2: 1x10^7 Tregs/kg; Cohort 3: 3x10^7 Tregs/kg | NCT00725062 | Phase I  Enrolment (actual): 3 participants  Recruitment status: terminated (slow accrual.) |
| Patients undergoing myeloablative allogeneic hematopoietic cell transplant transplantation (MA-alloHCT) for hematologic malignancies | T-cell-Depleted Graft With Additional Infusion of Conventional T Cells and Regulatory T Cells  Product acronym TregGraft: Orca-T  *Comment:* No specific dose communicated (clinicaltrials.gov) | NCT05316701 | Phase III randomized, open-label, multicentre study  Enrolment (estimated): 174 participants  Recruitment status: recruiting |
| Patients receiving mismatched related or mismatched unrelated unmanipulated donor HSCT for hematologic malignancies | Cell preparation from the same donor of the HSCT (T-allo10) containing T regulatory type 1 (Tr1) cells able to suppress allogenic (host-specific) responses  Cohort 1: 1x10^6/kg (± 10%)  Cohort 2: 3x10^6/kg (± 10%)  Cohort 3: 9x10^6/kg (± 10%) | NCT03198234 | Phase I non-randomized, open label trial  Enrolment (estimated): 30 participants  Recruitment status: recruiting |
| **Autoimmunity** | | | |
| Paediatric type I Diabetes mellitus | 10/20 million autologous 1 – 2 week expanded FACS sorted CD3+ CD4+ CD25high CD127− Tregs/kg 1 – 2 months after onset | (Marek-Trzonkowska et al., 2012) | Phase I/II  Enrolment (actual): 10 participants  Recruitment status: completed  Results: safe and tolerable, two patients independent from insulin after 4 – 5 months, improved plasma C-peptide levels; For detailed results, see published reference |
| Recent onset (3 – 24 months) type I Diabetes mellitus | Peripheral blood-derived 5/40/320 million CD4+ CD127_low_/- CD25+ flow cytometry-sorted autologous polyclonal Tregs | NCT01210664  (Bluestone et al., 2015; Gitelman & Bluestone, 2016) | Phase I/II  Enrolment (actual): 16 participants  Recruitment status: completed  Results: Good tolerability; stable beta cell function (up to 2 years) |
| Recent onset (3 – 24 months) type I Diabetes mellitus | Peripheral blood-derived 3/20 million/kg of CD4+ CD127_low_ /- CD25+ flow cytometry-sorted autologous polyclonal Tregs combined with 1 million IU of recombinant IL-2 (daily subcutaneous injection) | NCT02772679 | Phase I  Enrolment (actual): 16 participants  Recruitment status: completed |
| Autoimmune Diabetes mellitus diagnosed within last 3 years | 1-5 million/kg of umbilical cord-derived Tregs | NCT02932826 | Phase I/II  Enrolment (estimated): 40 participants  Recruitment status: recruiting |
| Autoimmune Diabetes mellitus | 1-5 million/kg of umbilical cord-derived Tregs combined with Liraglutide | NCT03011021 | Phase I/II  Enrolment (estimated): 40 participants  Recruitment status: recruiting |
| Recent onset type I Diabetes mellitus in adolescents | Autologous high or low dose of 2.5/20 million cells/kg *ex-vivo* expanded polyclonal Tregs | NCT02691247 | Phase II  Enrolment (actual): 113 participants  Recruitment status: completed  Results: well tolerated, no significant impact on C-peptide after one year |
| Autoimmune hepatitis | 10-20 millions/kg of autologous polyclonal CD4+ CD127- CD25+ Tregs | NCT02704338 | Phase I/II  Enrolment (estimated): 30 participants  Recruitment status: unknown |
| Lupus erythematosus | 100 million autologous CD4+ CD127_low_ /- CD25+ Tregs | NCT02428309  (Romano, Fanelli, Albany, Giganti, & Lombardi, 2019) (Dall’Era et al., 2019) | Phase I (Pilot)  Enrolment (actual): 1 participant  Recruitment status: terminated due to patient recruitment issues  Results: Highly activated Tregs in diseased skin, marked attenuation of the interferon‐γ pathway, augmentation of IL‐17 pathway |
| Pemphigus foliaceus/ vulgaris | Cohort 1: 1.0 x 10^8 autologous polyclonal Tregs  Cohort 2: 2.5x10^8 autologous polyclonal Tregs | NCT03239470 | Phase I  Enrolment (actual): 5 participants  Recruitment status: Active, not recruiting  Enrolment stopped early, on May 1, 2020, due to (a) lack of recruitment, (b) ongoing and new feasibility issues incl. recent approval of rituximab for treating pemphigus and (c) impact of COVID-19 pandemic. No participants enrolled in cohort 2. |
| Active Crohn’s disease | Intravenous autologous polyclonal CD4-enriched flow cytometry-sorted CD4+ CD25_high_ CD127_low_ CD45RA+ Tregs | NCT03185000  (Goldberg et al., 2019) | Phase I/II (First-in-human)  Enrolment (estimated): 24 participants  Recruitment status: unknown |
| Refractory Crohn's disease | single intravenous injection of 10^6^, 10^7^, 10^8^, or 10^9^ autologous ovalbumin-specific Treg | Eudract, Number: 2006-004712-44 (Desreumaux *et al*. 2012) | open-label, multicentre, single-injection, escalating-dose, phase I/IIa clinical study  20 patients  well tolerated, dose-related efficacy |
| Active Ulcerative Colitis | Autologous leukapheresis-derived intravenous 0.5/ 1/ 2/ 5/ 10 million CD4+CD25+CD127-/_low_ Tregs/kg expanded for 21 days | NCT04691232 | Phase I  Enrolment (estimated): 10 participants  Recruitment status: recruiting |
| Refractory lupus nephritis; Type 1 diabetes | Autologous adoptive T regulatory cell transfer (CD4+, CD25+, CD127-, FoxP3+)  *Comment:* No specific dose communicated (clinicaltrials.gov) | NCT05566977 | Phase I non-randomized open-label  Enrolment (estimated): 20 participants  Recruitment status: not yet recruiting |
| Islet Transplantation | Cryopreserved polyclonal regulatory T Cells (cePolyTregs);  Target 400-1600 million, with a minimal acceptable dose of 100 million) two weeks post islet transplant | NCT05349591 | Phase I non-randomized open-label  Enrolment (estimated): 11 participants  Recruitment status: recruiting |
| **Other** | | | |
| Early and late-stage Amyotrophic Lateral Sclerosis | 4x (2 [early] – 4 month [late]) leukapheresis-derived autologous 1 million/kg intravenous CliniMACS CD19/CD8-depleted CD25-enriched Tregs combined with 3x per week 2 × 10^5^ IU/m^2^ subcutaneous IL-2 | NCT03241784  (Alsuliman et al., 2016; Thonhoff et al., 2018) | Phase I/II  Enrolment (estimated): 4 participants  Recruitment status: unknown  Preliminary results: Well tolerated, safe, slowed disease progression |
| Amyotrophic Lateral Sclerosis | 6x monthly leukapheresis-derived autologous intravenous CliniMACS CD19/CD8-depleted CD25-enriched Tregs combined with 3x per week low-dose subcutaneous IL-2 | NCT04055623 | Phase IIa  Enrolment (estimated): 12 participants  Recruitment status: active, not recruiting |
| Amyotrophic Lateral Sclerosis | Autologous hybrid Treg/Th2 cell product expanded for 7 days | NCT04220190 | Phase I/II  Enrolment (estimated): 21 participants  Recruitment status: recruiting |
| Liver cirrhosis | Tregs combined with mesenchymal stem cells | NCT03460795 | Phase I/II trial  Enrolment (estimated): 30 participants  Recruitment status: Not yet recruiting |
| Mild to moderate Alzheimer’s disease | 170,000/kg intravenous autologous Tregs (other name: GB301) | NCT03865017 | Phase I  Enrolment (estimated): 20 participants  Recruitment status: unknown |
| COVID-19 induced acute respiratory distress syndrome | 100/300 million cryopreserved off-the-shelf cord blood-derived Tregs expressing lung-homing markers | NCT04468971 | Phase I/II (safety and early efficacy)  Enrolment (actual): 45 participants  Recruitment status: completed  No results posted yet (clinicaltrials.gov registry) |
| COVID-19 induced acute respiratory distress syndrome | 40/160 million allogenic cryopreserved off-the-shelf hybrid Treg/TH2 cells expanded for 7 days | NCT04482699 | Phase I/IIb  Enrolment (estimated): 88 participants  Enrolment (actual): 1 participant  Recruitment status: Terminated due to change in eligible patient population |
| Alzheimer's Disease  (Mild-to-Moderate) | off-white suspension of Tregs (1.7x10^5 cells/kg±15%) for injection diluted with sterile saline solution and supplied in clear, colourless, polypropylene vials (VT301)  low dose: 8.5x10^4 cells/kg vs. high dose: 1.7x10^5 cells/kg | NCT05016427 | Phase I  Enrolment (estimated): 12 participants  Recruitment status: recruiting |
| Haematological Malignancies | UCB Tregs and CD3+ Teff cells  dose escalation: 3x10^6/kg, 10x10^6/kg, 30x10^6/kg, 100x10^6/kg, 300x10^6/kg | NCT01163201 | Phase I/II  Enrolment (actual): 0 participants  Recruitment status: withdrawn (replaced by a new study) |
| relapsed/refractory (R/R) CD19+ B Acute Lymphocytic Leukaemia (B-ALL) | allogeneic CAR19 regulatory T cells (CAR19-tTreg), single dose, dose escalation: 1x10^6 cells/kg bw, 3x10^6 cells/kg bw, 10x10^6 cells/kg bw, 30x10^6 cells/kg bw  Other products: Fludarabine, Cyclophosphamide | NCT05114837 | Phase I/II  Enrolment (estimated): 31 participants  Recruitment status: not yet recruiting |

***Abbreviations:*** ATG - anti-thymocyte globulin, bw – body weight, CAR – chimeric antigen receptor, CD – cluster of differentiation, CNI – calcineurin inhibitors, COVID19 - coronavirus disease 2019, d – day, FACS – Fluorescence activated cell sorting, FoxP3 - Forkhead Box P3, GCSF - granulocyte colony-stimulating factor, GvHD – graft versus host disease, HCT – hematopoietic cell transplantation, hi – high, HLA – human leukocyte antigen, HSCT – hematopoietic stem cell transplantation, ID – identifier, IL – interleukin, IU – international unit, kg – kilogram, MRD – matched related donor, mTOR - mammalian Target of Rapamycin, MUD – matched unrelated donor, NA – not applicable, PB – peripheral blood, Tconv(s) – conventional T cell(s), Th – T helper, Treg(s) – regulatory T cell(s), UCB – umbilical cord blood.

Registry summary search strategy:

50 Studies found for: **Regulatory T cell | kidney transplant**

Also searched for **Transplantation**, **Kidney Transplantation**, **Renal transplant** and more

74 Studies found for: **Regulatory T cell | liver**

Also searched for **T reg**, **T-regulatory cell**, **Regulations** and more.

54 Studies found for: **Regulatory T cell | GvHD**

Also searched for **Graft Versus Host Disease**, **Graft vs Host Disease**, **T reg** and more

84 Studies found for: **Regulatory T cell | HSCT**

Also searched for **Stem Cell Transplantation**, **Stem Cell Transplant**, **Hematopoietic Cell Transplantation** and more

199 Studies found for: **Regulatory T cell | autoimmunity**

Also searched for **Autoimmune disease**, **T reg**, **T-regulatory cell** and more.

106 Studies found for: **Regulatory T cell | intestine**

Also searched for **Intestinal**, **T reg**, **T-regulatory cell** and more

107 Studies found for: **Regulatory T cell | neurologic**

Also searched for **Nervous System**, **T reg**, **T-regulatory cell** and more.

587 Studies found for: **Regulatory T cell | cancer**

Also searched for **Neoplasm**, **T reg**, **T-regulatory cell** and more.
